# P-226. GLP-1 Use and Outcomes in Persons with and without HIV

**DOI:** 10.1093/ofid/ofaf695.448

**Published:** 2026-01-11

**Authors:** Cassidy Boomsma, Jacob Sinkowitz, Angela T Perez Villalobos, Sainabou Jallow, Rajeev Agrawal, Amanda B Spence

**Affiliations:** Medstar Georgetown University Hospital, Washington, DC; Georgetown University School of Medicine, Washington DC, District of Columbia; Georgetown University School of Medicine, Washington DC, District of Columbia; Georgetown University School of Medicine, Washington DC, District of Columbia; Medstar Georgetown University Hospital, Washington, DC; Georgetown University Medical Center, District of Columbia, District of Columbia

## Abstract

**Background:**

Glucagon-like peptide-1 (GLP-1) agonists are approved for the treatment of diabetes and obesity. Their benefits include cardiovascular risk reduction, renal protection, and potential prevention of cognitive dysfunction. Early clinical trials in persons with HIV (PWH) showed improvements in lipohypertrophy and steatotic liver disease. Given the high burden of cardiometabolic comorbidities among PWH, GLP-1 receptor agonists may offer significant health benefits. Evaluation of real-world GLP-1 medication use and efficacy in PWH is needed.

**Figure:**
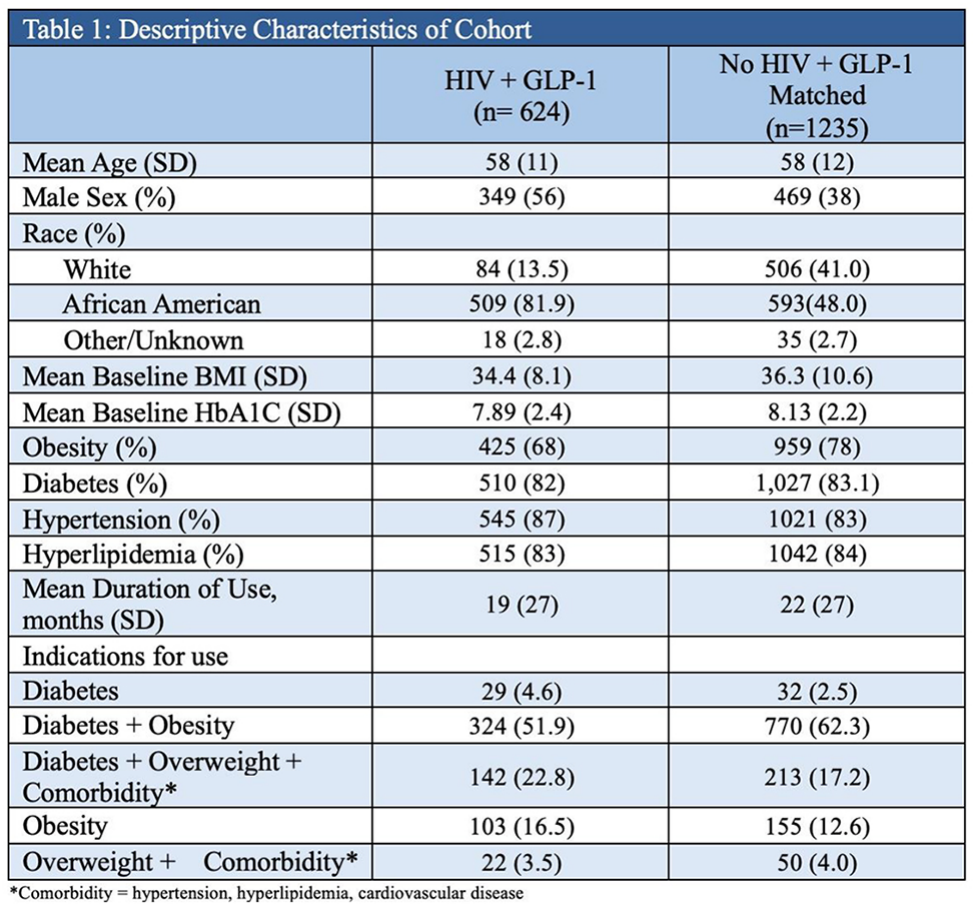

**Figure:**
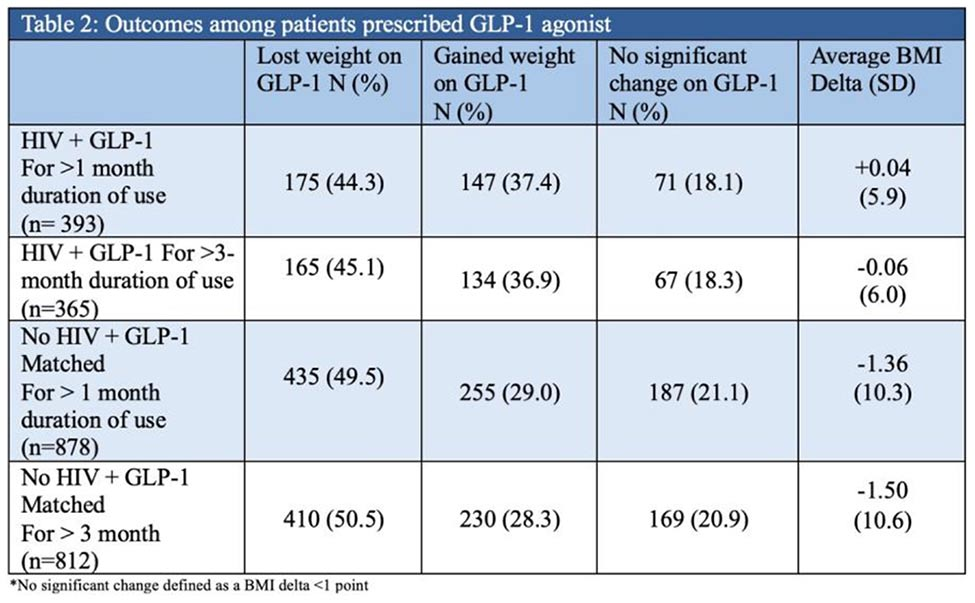

**Methods:**

Using electronic health record data, we identified all PWH seen at a MedStar Health infectious disease or primary care clinic between 2013 – 2024 who were prescribed a GLP-1. PWH were age-matched (2:1) to individuals without HIV. Demographics, HIV status, body mass index (BMI), hemoglobin A1C (HbA1c), comorbidities (e.g., hypertension, diabetes, and hyperlipidemia), and GLP-1 type and duration of use were extracted. We compared GLP-1 indications and health outcomes by HIV status.

**Results:**

A total of 625 PWH and 1,235 age-matched persons without HIV were included. The mean age was 58 years (SD 11.8). The PWH had higher proportion of males (56% vs. 38%) and African American population (82% vs. 48%). Baseline BMI, HbA1c, and prevalence of diabetes, hypertension, and hyperlipidemia were similar across groups. However, PWH had lower prevalence of obesity (68% vs 78%) and shorter mean duration of GLP-1 use (19 vs. 22 months). Initial review of PWH using GLP-1 from 2020-2024 (n=91) indicates the most commonly prescribed GLP-1 is semaglutide injection (59%). Among patients with >3 months of GLP-1 use, a smaller proportion of PWH experienced a BMI reduction (45.1% vs. 50.5%), with a lower mean BMI change (–0.06 vs. –1.5).

**Conclusion:**

Persons with and without HIV had similar comorbidities, but differences in demographics and outcomes require further assessment. Ongoing studies in this population are needed to evaluate medication access, tolerance, and long-term cardiometabolic outcomes.

**Disclosures:**

All Authors: No reported disclosures

